# Special Hospitals

**Published:** 1921-08-27

**Authors:** 


### Special Hospitals.

**Central London Throat and Ear Hospital, Gray's Inn Road, W.C.**—Three courses of instruction are open to practitioners attending the hospital: first, the course in methods of examination and diagnosis; second, the course of systematic instruction in the diseases of the nose, throat, and ear; and third, the operative surgery class. The course in methods of examination and diagnosis is introductory in character. It comprises four lessons of practical teaching in the actual examination of patients and in the manipulation of instruments. Clinical assistants, especially if they have not had any previous experience in the speciality, are expected to attend this class in order to become acquainted with the minutiæ of the methods of diagnosis, etc. The class is free to clinical assistants. To ordinary students and practitioners the fee is one guinea. Intending students (who can join at any time) are requested to give their names to the Dean. Attendance at this class is compulsory for those who desire to obtain the higher-grade certificate. The second course of systematic instruction in diseases is more advanced. It consists of over thirty lessons in all on pathology, diagnosis, and treatment. Full syllabus may be had on application to the Secretary.

**Hospital for Consumption and Diseases of the Chest, Brompton, S.W.**—Students may attend this hospital at a fee of £2 2s. for one month, £4 4s. for three months, or £7 7s. for six months. Demonstrations are given in the wards, out-patient department, *x*-ray and throat departments. Clinical assistants are appointed to the physicians and assistant physicians. For particulars apply to the Dean, Dr. L. S. Burrell.

**Hospital for Diseases of the Throat Golden Square, London, W.,** with which is amalgamated the London Throat Hospital, Great Portland Street.—The hospital contains sixty beds. There is an out-patient attendance of over 60,000 yearly. Clinical instruction in the diagnosis and treatment of disease is given in the out-patient department from 2 to 5 P.M. on Tuesdays, and Fridays from 6.30 to 9 P.M. From amongst the students junior clinical assistants are selected, whose duty it is to assist the member of the staff to whom they are appointed. For terms and further information apply to the Dean, Mr. Lionel Colledge.

**The Hospital for Sick Children, Great Ormond Street, W.C.**—This school is one of the post-graduate teaching schools of the University of London, and is recognised by the Conjoint Board of England and by the Universities of London, Oxford, and Cambridge, as a place where undergraduates may spend six months of their fifth year in clinical work, or hold the appointments of clinical clerks and dressers. There are 240 beds, to which nearly 3,000 cases are admitted per annum, and in the out-patient department the average number of new cases each year exceeds 25,000. Clinical instruction is given daily in the wards, out-patient department, and operating theatre by members of the visiting staff. Fees for one month's attendance, two guineas; for three months, five guineas; perpetual ticket, ten guineas; clinical clerk's reduced fee of £1 1s. for one month. A certificate is given at the end of a three months' course. Further details and full syllabus may be obtained by application to the Dean, Sub-Dean, or the Secretary of the hospital. A limited number of clerkships in the pathological department, affording facilities for theoretical and practical instruction in clinical pathology and bacteriology suitable for post-graduates and fifth-year students, are available. Fees for one month's course, three guineas; two months' course, five guineas; three months' course, six guineas. For detailed syllabus application should be made to the Dean or the Secretary. The Dean is Mr. O. L. Addison and the Sub-Dean Dr. W. J. Pearson, from whom all further information can be obtained, or from the Secretary at the hospital.

**The Hospital for Women, Soho Square, W.** The teaching in connection with the above hospital has been greatly extended, and is available for post-graduates only in limited numbers. Every facility is afforded them by the teaching staff for obtaining experience in the diagnosis and treatment of gynæcological diseases. The hospital contains sixty-eight beds, and the out-patient attendances are over 14,000 per annum. Operations take place daily at two o'clock, except on Saturdays. The *x*-ray department is supplied with the latest installation both for diagnosis and treatment, and experience can also be gained in the treatment of malignant disease by radium. Incorporated with the above hospital is the London School of Gynæcology, which has three sessions in the year, each of eight weeks' duration, comprising attendance in out-patients' department, operating theatre, clinical lectures, demonstrations in pathology, and *x*-ray treatment. For syllabus and particulars apply to the Dean, Sidney Forsdike, M.D., F.R.C.S., Hospital for Women, Soho Square.

**National Hospital for the Paralysed and Epileptic, Queen Square, W.C.**— The in- and out-patient practice of this hospital is open at 2 P.M. every day (except Wednesday and Saturday). Patients are seen by the physicians, the physicians for out-patients, and the assistant physicians. Three courses of lectures and clinical demonstrations are given in each year. The fee for out-patient hospital practice and lectures is five guineas for three months, seven guineas for six months, and ten guineas for a perpetual ticket; for in-patient clerking a fee of five guineas for three months or seven guineas for six months is charged. The museum is available for the prosecution of special work under the pathologist, for which a separate fee is charged. Women students attend the practice of the hospital. All communications should be sent to the Dean of the Medical School.

**Ophthalmic Hospitals.**—Ophthalmic hospitals are the Central London Ophthalmic Hospital, Judd Street, W.C.; the Royal London Ophthalmic Hospital, City Road, E.C.; the Royal Eye Hospital, St. George's Circus, S.E.; and the Royal Westminster Ophthalmic Hospital, King William Street, W.C. In each of these the fees charged are very modest.

**Queen Charlotte's Lying-in Hospital.**— Qualified medical practitioners and medical students are admitted to the practice of this hospital. Last year (1920) there were 1,812 women delivered in the wards, about one-half being primiparæ. A large number of the cases were abnormal. In the ante-natal department 4,860 patients attended for examination and treatment. Certificates of attendance at this hospital are recognised by all Universities, Colleges, and licensing bodies. Fee for the course of four weeks, £8 8s. Students are accommodated at the new Residential College (5 Cosway Street), opposite the hospital. This instruction includes (1) Practical instruction in the methods of examination of pregnant women; (2) delivery of women in labour under the direct supervision of a medical officer of the hospital; (3) practical instruction in the treatment of the mother and child during the puerperium, including clinics held four times weekly by the visiting medical staff; (4) instruction in the clinical laboratory of the hospital.

**Royal Hospital for Diseases of the Chest, City Road, E.C.**—The departments in which study can be pursued comprise the in-patient, out-patient, Röntgen-ray, cardiological, pathological and bacteriological, ear, nose and throat, and dental departments. An extensive department for the prevention of consumption (tuberculosis dispensary) has been established for some years. As this works in connection with three large boroughs—namely, Shoreditch, Finsbury, and Islington—a splendid opportunity presents itself for medical practitioners and students who desire to obtain an intimate knowledge of this work. Attached to the hospital is a medical school for instruction in diseases of the chest, more especially tuberculosis, at which courses of instruction are given from time to time. There is also a tuberculosis school for nurses, where regular theoretical and thoroughly practical courses of instruction are given, for which a certificate is granted. The fees for attending the clinical practice of the hospital are as follows: One month, one guinea; three months, three guineas; six months, five guineas; perpetual ticket, six guineas. Any further information will be gladly given by the Dean.

**St. John's Hospital for Diseases of the Skin.**— Out-patient Department, 49 Leicester Square, W.C. 2; In-patient Department, 262 Uxbridge Road, W. This hospital has a well-equipped in-patient department, with forty beds. It has a School of Dermatology at 49 Leicester Square, which is conducted by the medical staff of the hospital. During the winter the free course of Chesterfield lectures is given on Thursday evenings at six o'clock by Drs. Wm. Griffith, J. L. Bunch, and Knowsley Sibley, and is always well attended by the profession. At the end of the course the "Chesterfield" Silver Medal may be competed for. The opening lecture will be given by Dr. Griffith on Thursday, October 6. The out-patient department contains a spacious laboratory, and an electrical department recently enlarged. Special courses in the pathology and bacteriology of the skin may be arranged for. The daily clinics at 2, and every evening (except Saturday) at 6, are open to practitioners upon signing the Visitors' Book. Venereal diseases, under the Government scheme, are seen at all clinics.

